# Supplementary material for: Evaluating Polarizable Biomembrane Simulations against Experiments
Source: J Chem Theory Comput. 2024 May 8;20(10):4325–37. doi: 10.1021/acs.jctc.3c01333 (PMC11137822; doi:10.1021/acs.jctc.3c01333)
Supplement: Supplementary file 1 — ct3c01333_si_001.pdf [file ct3c01333_si_001.pdf]

Supplementary Information: Evaluating polarizable biomembrane simulations against experiments

Hanne S. Antila,[*,†,‡,¶] Sneha Dixit,[†] Batuhan Kav,[*,§] Jesper J. Madsen,[‖,⊥] Markus S. Miettinen,[†,#,¶] and O. H. Samuli Ollila[@,△]

†*Department of Theory and Bio-Systems, Max Planck Institute of Colloids and Interfaces, 14476 Potsdam, Germany*

‡*Department of Biomedicine, University of Bergen, 5020 Bergen, Norway*

¶*Computational Biology Unit, Department of Informatics, University of Bergen, 5008 Bergen, Norway*

§*Institute of Biological Information Processing: Structural Biochemistry (IBI-7), Forschungszentrum Jülich, 52428 Jülich, Germany*

‖*Department of Molecular Medicine, Morsani College of Medicine, University of South Florida, Tampa, Florida 33612, United States of America*

⊥*Center for Global Health and Infectious Diseases Research, Global and Planetary Health, College of Public Health, University of South Florida, Tampa, Florida 33612, United States of America*

#*Department of Chemistry, University of Bergen, 5007 Bergen, Norway*

@*VTT Technical Research Centre of Finland, 02044 Espoo, Finland*

△*Institute of Biotechnology, University of Helsinki, 00014 Helsinki, Finland*

E-mail: hanne.antila@uib.no; batuhankav@gmail.com

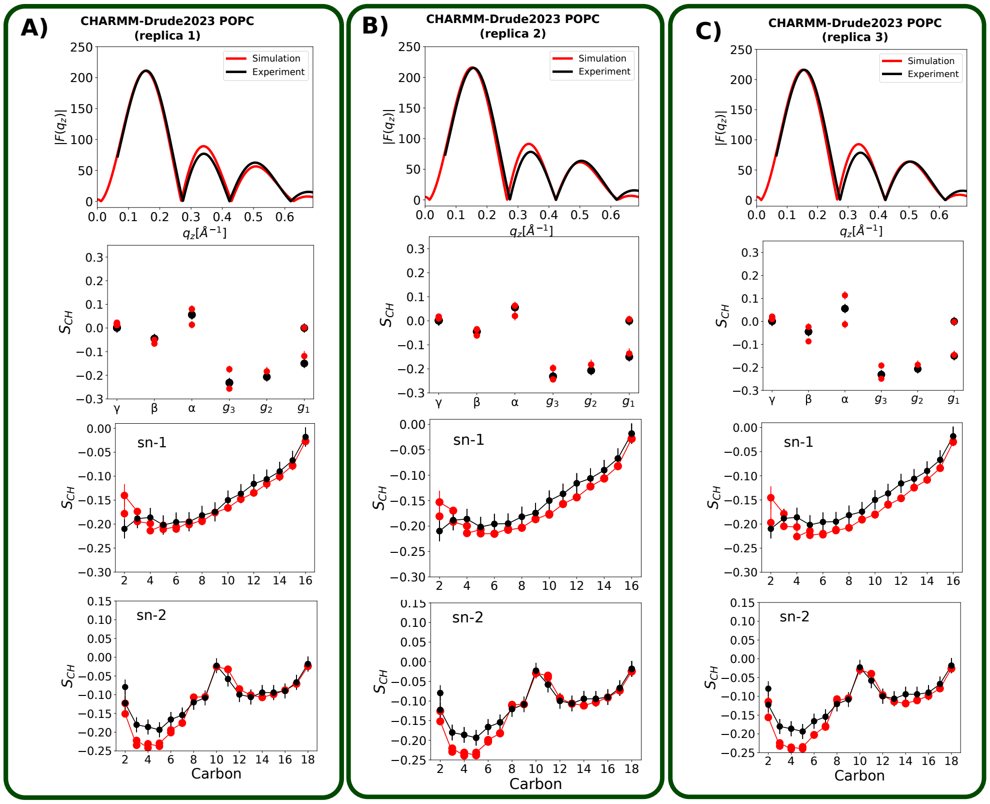

Figure S1: Comparison of different replicas from CHARMM-Drude2023 simulations against experimental X-ray scattering form factors and C–H bond order parameters from the NMRlipids Databank.

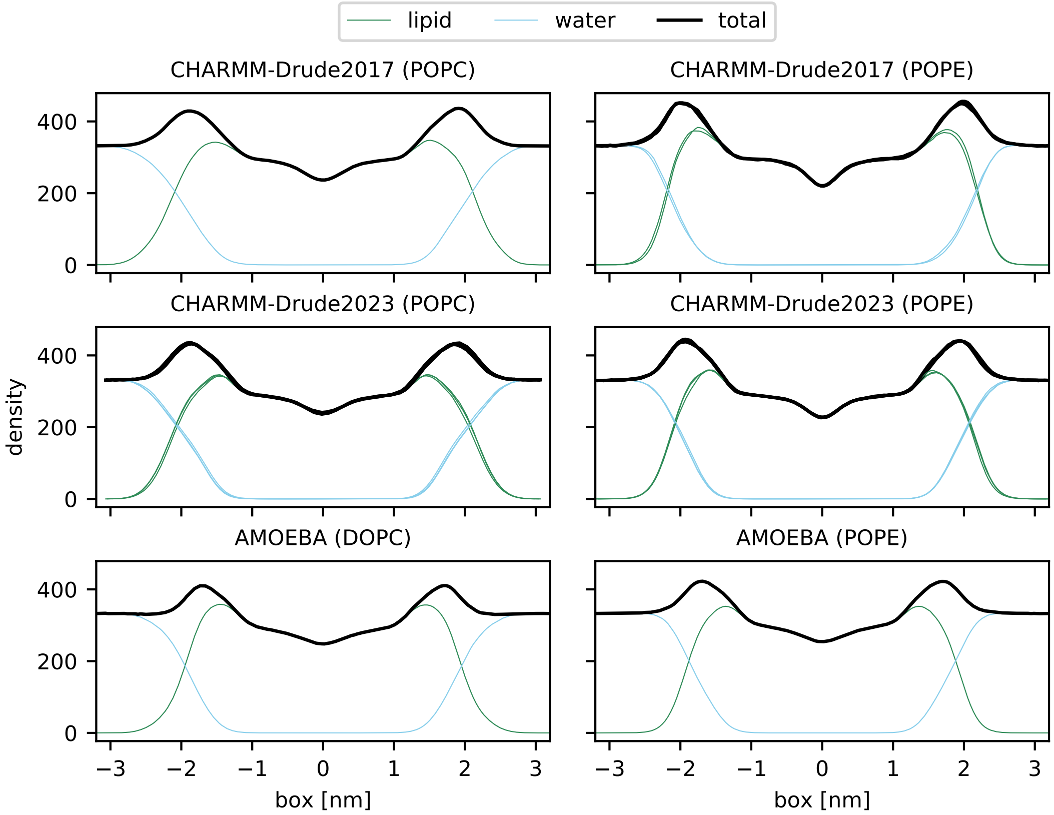

Figure S2: The bilayer electron density profiles from salt-free simulations with the investigated polarizable models. The data are centered such that the bilayer center resides at zero. For some systems, multiple replicas are plotted as indicated by the presence of multiple (mostly overlapping) lines.

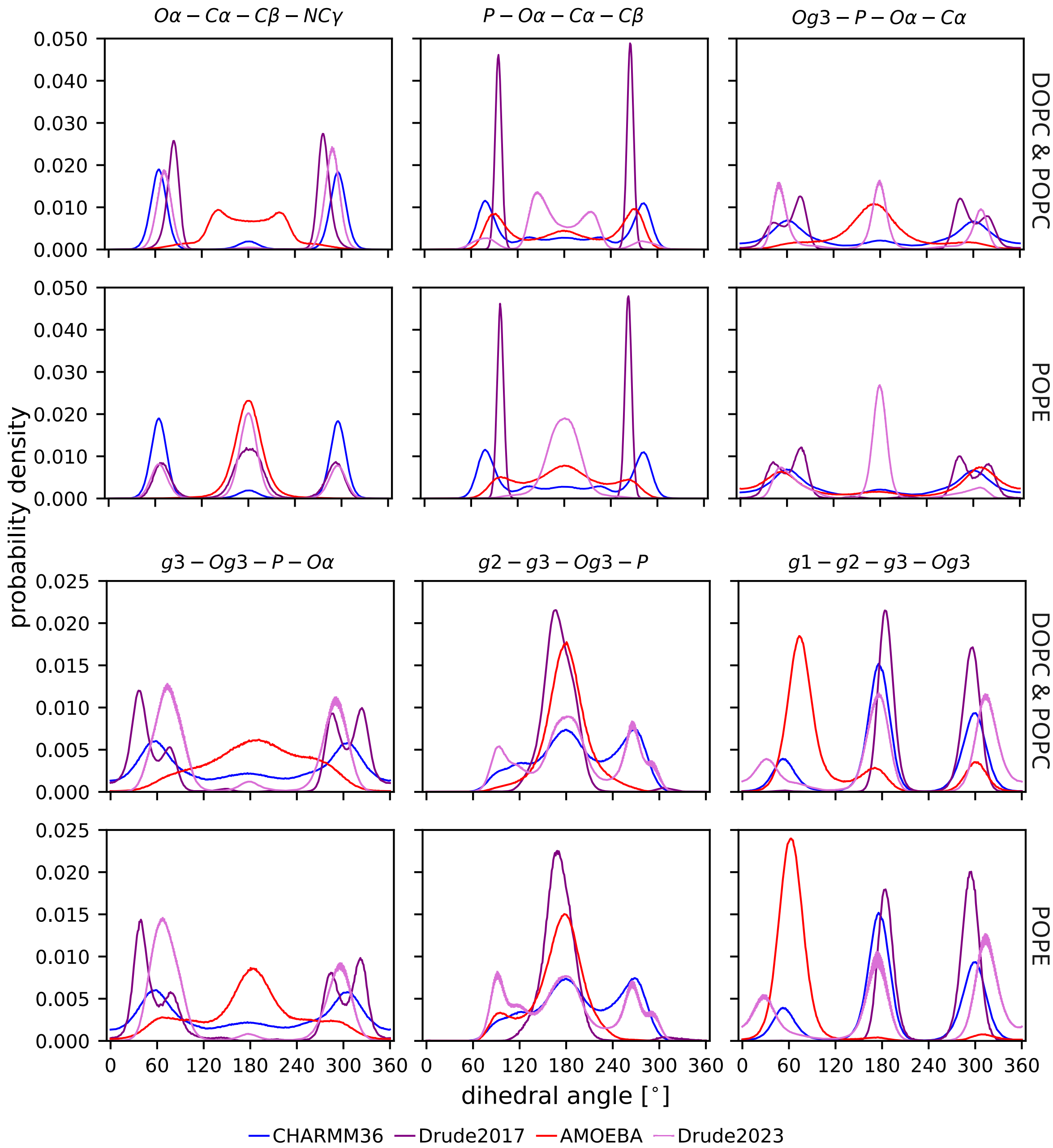

Figure S3: Distributions of the torsion angles for head group atoms. The atoms forming the dihedral angles are given in the titles. For each torsion angle, the upper rows contain DOPC (AMOEBA) and POPC (CHARMM-Drude2017/CHARMM-Drude2023) data and the lower rows contain dihedrals for POPE.

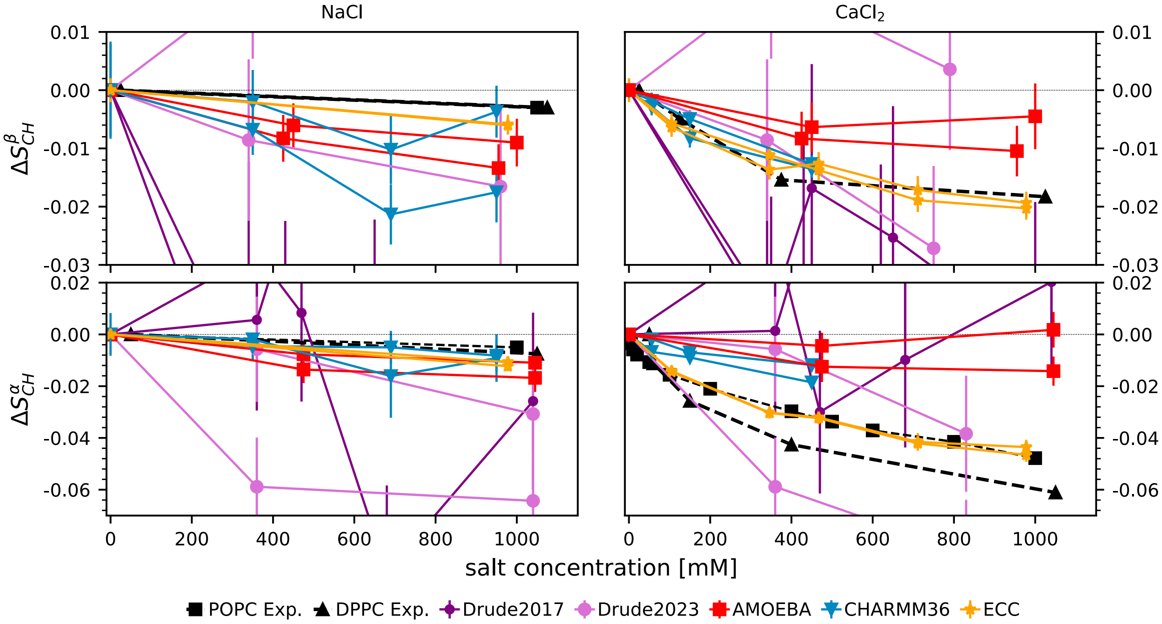

Figure S4: The change in the lipid head group order parameters β (top row) and α (bottom row) upon increasing ion concentration with respect to the simulations without salt. Data plotted separately for the two hydrogens attached to each carbon. CHARMM36 and ECClipids data are reproduced using the Zenodo repositories at Refs. 1–4 and Ref. 5, respectively.

References

(1) Ollila, S. MD simulation trajectory and related files for POPC bilayer with 350mM NaCl (CHARMM36, Gromacs 4.5). 2015; https://doi.org/10.5281/zenodo.32496.

(2) Ollila, S. MD simulation trajectory and related files for POPC bilayer with 690mM NaCl (CHARMM36, Gromacs 4.5). 2015; https://doi.org/10.5281/zenodo.32497.

(3) Ollila, S. MD simulation trajectory and related files for POPC bilayer with 950mM NaCl (CHARMM36, Gromacs 4.5). 2015; https://doi.org/10.5281/zenodo.32498.

(4) Nencini, R. CHARMM36, NB-Fix approaches, without NBFIX, POPC membrane, Ca, Na ions,. 2019; https://doi.org/10.5281/zenodo.3434396.

(5) Melcr, J. Simulations of POPC lipid bilayer in water solution at various NaCl, KCl and CaCl2 concentrations using ECC-POPC force field. 2017; https://doi.org/10.5281/zenodo.3335503.
